# Distinct macular structural and microvascular alterations differentiate neuromyelitis optica spectrum disorder from myelin oligodendrocyte glycoprotein antibody–associated disease in optic neuritis

**DOI:** 10.3389/fimmu.2026.1759144

**Published:** 2026-03-10

**Authors:** Mi Zhang, Zhongzhong Liu, Yunfei Li, Yanli Li, Ruili Ma, Qingli Lu, Pei Liu, Yan liu, Qiaoqiao Chang, Yan Wang, Chensheng Song, Yan Huo, Lanping Rao, Shundao Cao, Ning Wang, Guo Li, Fanyan Wu, Tong Liu, Linna Peng, Yunlong Hao, Zijing Cao, Xuemei Lin, Xiaolai Zhou, Songdi Wu

**Affiliations:** 1 Department of Neuro-ophthalmology, Xi’an No.1 Hospital, The First Affiliated Hospital of Northwest University, Xi’an, Shaanxi, China; 2 Xi’an Key Laboratory for Innovation and Transformation of Neuroimmunological Diseases, Xi’an, Shaanxi, China; 3 Department of Epidemiology and Biostatistics, School of Public Health of Xi’an Jiaotong University Health Science Center, Xi’an, Shaanxi, China; 4 Department of Sensory Control, Xi’an No.1 Hospital, The First Affiliated Hospital of Northwest University, Xi’an, Shaanxi, China; 5 Key Laboratory of Resource Biology and Biotechnology in Western China, Ministry of Education, School of Medicine, Northwest University, Xi’an, Shaanxi, China; 6 School of Medicine, Xizang Minzu University, Xianyang, Shaanxi, China; 7 State Key Laboratory of Ophthalmology, Zhongshan Ophthalmic Center, Sun Yat-Sen University, Guangzhou, Guangdong, China; 8 Guangdong Provincial Key Laboratory of Ophthalmology and Visual Science, Sun Yat-Sen University, Guangzhou, Guangdong, China; 9 Guangdong Basic Research Center of Excellence for Major Blinding Eye Diseases Prevention and Treatment, Sun Yat-Sen University, Guangzhou, Guangdong, China

**Keywords:** macular microvasculature, myelin oligodendrocyte glycoprotein antibody-associated disease, neuromyelitis optica spectrum disorder, optic neuritis, optical coherence tomography angiography, retinal biomarkers

## Abstract

**Introduction:**

Neuromyelitis optica spectrum disorder (NMOSD) and myelin oligodendrocyte glycoprotein antibody–associated disease (MOGAD) are among the leading causes of optic neuritis. This study aimed to examine differences in macular retinal structure and microvascular characteristics between affected and unaffected eyes in individuals with NMOSD and MOGAD.

**Method:**

This cross-sectional study enrolled both eyes of patients diagnosed with optic neuritis (ON)secondary to NMOSD (22 patients: 36 NMOSD-ON eyes and 8 NMOSD-NON eyes), MOGAD (23 patients: 34 MOG-ON eyes and 12 MOG-NON eyes), and 20 age- and sex-matched healthy controls (HCs, 40 eyes) recruited from the First Affiliated Hospital of Northwest University (Xi’an No.1 Hospital) between February 2023 and January 2025. Microvascular density (MVD), vascular density (VD), blood flow area (BFA), and macular ganglion cell–inner plexiform layer (GCIPL) thickness were measured and analyzed.

**Result:**

Both NMOSD-ON and MOG-ON eyes showed significant reductions in MVD of radial peripapillary capillary plexus (RPCP); MVD, VD, and BFA of superficial vascular complex (SVC); BFA of deep vascular complex (DVC); and GCIPL thickness compared with HCs (*P* < 0.001). Compared with MOG-ON eyes, NMOSD-ON eyes demonstrated a greater reduction in BFA of choriocapillaris (CC) (*P* = 0.040). In MOG-NON eyes, the MVD of RPCP; the MVD, VD, and BFA of SVC; the BFA of DVC; and the GCIPL thickness were significantly lower than those in HCs, but remained higher than in MOG-ON eyes. In NMOSD-ON eyes, all MVD and VD parameters, SVC BFA, and GCIPL thickness were inversely correlated with best-corrected visual acuity (BCVA) and Expanded Disability Status Scale (EDSS) scores (*P* < 0.05). In MOG-ON eyes, SVC MVD, VD, and GCIPL thickness were inversely correlated with BCVA and disease duration, while RPCP MVD and SVC BFA were inversely correlated only with BCVA.

**Conclusion:**

Both NMOSD and MOGAD cause macular structural and microvascular damage associated with reduced BCVA. Decreased CC BFA may aid in distinguishing NMOSD from MOGAD.

## 1 Introduction

Neuromyelitis optica spectrum disorder (NMOSD) is a rare autoimmune condition characterized by recurrent episodes of acute optic neuritis (ON) and transverse myelitis, which constitute its defining clinical features (1). The majority of individuals harbor immunoglobulin G (IgG) autoantibodies targeting aquaporin-4 (AQP4), although approximately 20–30% remain seronegative for AQP4-IgG (1, 2). With the identification of myelin oligodendrocyte glycoprotein antibody-associated disease (MOGAD), serological studies have shown that 9–42% of those seronegative for AQP4-IgG are positive for MOG-IgG (3) (4)., NMOSD and MOGAD differ markedly in pathogenesis, clinical presentation, treatment response, and prognosis (3, 5, 6). MOGAD may occur as a monophasic or relapsing disorder, whereas NMOSD typically presents as a relapsing condition. After acute treatment of ON episodes, visual function in most MOGAD cases usually recovers to near-normal levels, whereas NMOSD more commonly results in lasting visual impairment (6). Pathologically, MOGAD primarily involves MOG-IgG-mediated demyelination of oligodendrocytes, whereas NMOSD predominantly targets astrocytes (6, 7).

Optical coherence tomography angiography (OCTA) has recently become a widely used retinal vascular imaging modality in ophthalmic and neuro-ophthalmic practice. It enables rapid, noninvasive acquisition of high-resolution, depth-resolved data on retinal and choroidal perfusion, along with quantitative analysis of microvascular parameters (8). Previous studies have shown that ON leads to marked degeneration of the peripapillary retinal nerve fiber layer (RNFL), ganglion cell layer (GCL), and inner plexiform layer (IPL), as well as to retinal microvascular alterations (6, 9).However, comparative data on OCTA parameters between NMOSD and MOGAD remain limited. This study therefore aims to delineate differences in macular structure and microvascular characteristics, assessed by optical coherence tomography (OCT) and OCTA, between affected and unaffected eyes in individuals with NMOSD and MOGAD.

## 2 Materials and methods

### 2.1 Study design and participants

**Figure 1:**
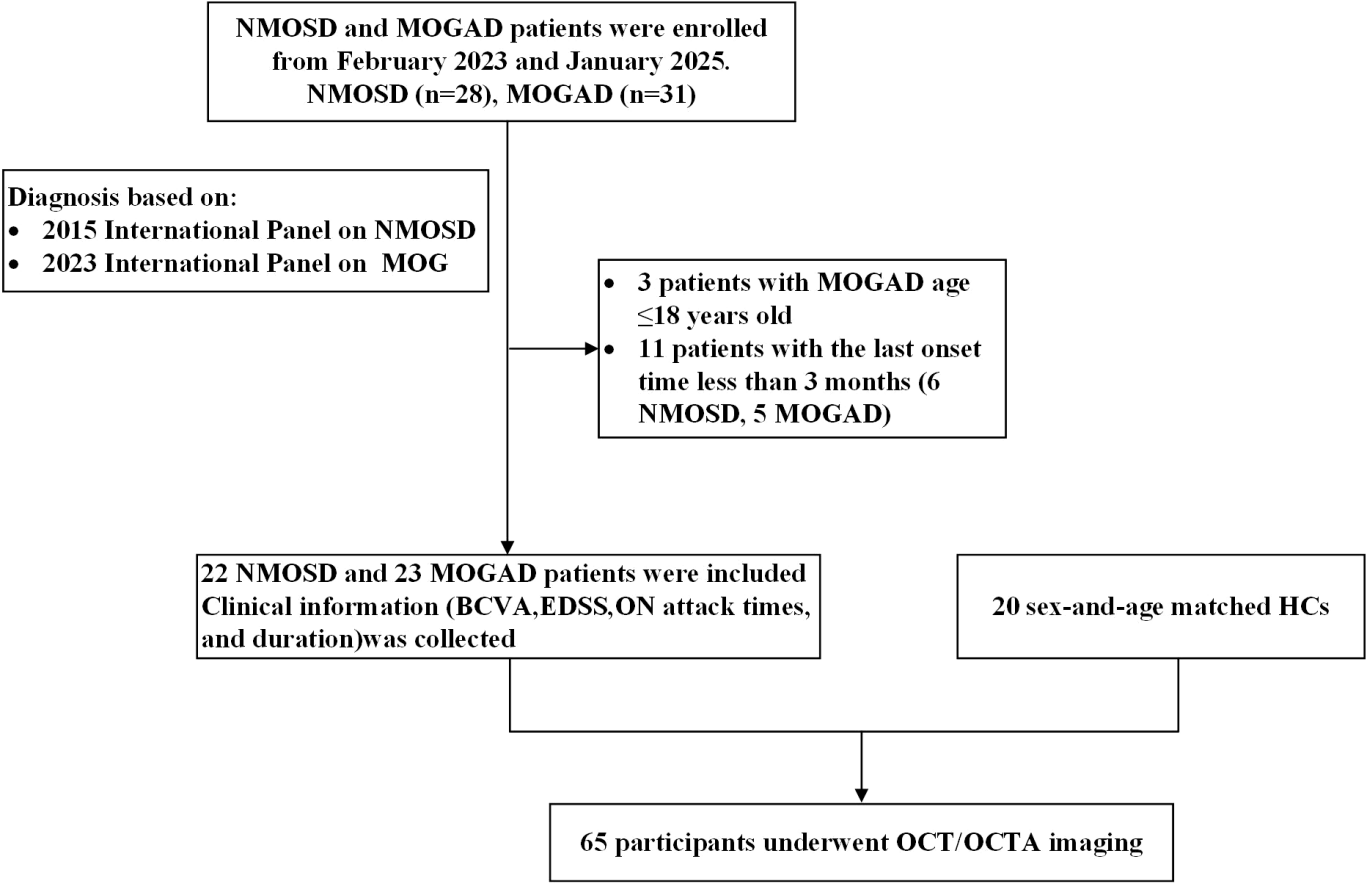
Flowchart of participant selection in this study. NMOSD, neuromyelitis optica spectrum disorder; MOGAD, myelin oligodendrocyte glycoprotein antibody–associated disease; HCs, healthy controls; BCVA, best-corrected visual acuity; EDSS, Expanded Disability Status Scale; OCT, Optical coherence tomography; OCTA, Optical coherence tomography angiography.

In this retrospective, observational, ross-sectional study, 45 individuals with a clinical diagnosis of optic neuritis (ON) were enrolled. Among them, 22 participants met the international consensus diagnostic criteria for neuromyelitis optica spectrum disorder (NMOSD) and were all seropositive for AQP4-IgG (10), and 23 participants met the diagnostic criteria for myelin oligodendrocyte glycoprotein antibody–associated disease (MOGAD) (11). Additionally, 20 age- and sex-matched healthy controls (HCs) were recruited. The participant enrollment and selection process are summarized in **Figure 1**.

**Figure 1** The flowchart of the participants in our study. NMOSD, neuromyelitis optica spectrum disorder; MOG, myelin oligodendrocyte glycoprotein; HCs, healthy controls; ON, optic neuritis; BCVA, best-corrected visual acuity; EDSS, Expanded Disability Status Scale; OCT, Optical coherence tomography; OCTA, Optical coherence tomography angiography.

All participants were consecutively enrolled at the Neuro-ophthalmology Clinic of the First Affiliated Hospital of Northwest University (Xi’an No.1 Hospital) between February 2023 and January 2025. The study was approved by the Institutional Ethics Committee of the First Affiliated Hospital of Northwest University (Xi’an No.1 Hospital), and written informed consent was obtained from all participants.

The inclusion criteria for patients were as follows: (1) age ≥18 years, in order to avoid potential statistical bias due to physiological differences in fundus characteristics between adolescents and adults; (2)intraocular pressure ≤ 21 mmHg and spherical equivalent refractive error within ±3.00 D; (3) interval of more than 3 months since the last ON episode (12); (4) seropositivity for MOG-IgG or AQP4-IgG as determined by cell-based assay (CBA); and (5) ability to fixate during OCT and OCTA examinations and cooperate with the examiner.

Exclusion criteria for all participants included: (1) ocular conditions that could significantly affect OCTA measurements, such as moderate -to -severe cataracts, glaucoma, retinal vascular disease, macular degeneration, epiretinal membrane, ocular trauma, or prior intraocular surgery; or systemic disorders that might confound results, including uncontrolled hypertension, uncontrolled diabetes mellitus, hematologic diseases, or vasculitis; (2) inability to cooperate with OCTA or acquisition of poor-quality images precluding reliable data analysis; and (3) refusal to participate or failure to provide written informed consent.

All participants underwent comprehensive systemic and neurological evaluations. Clinical data were recorded, including diagnosis, number of ON episodes and disease history for each eye, disease duration, and Expanded Disability Status Scale (EDSS) scores. Neuro-ophthalmologic assessments were performed for all enrolled individuals, including best-corrected visual acuity (BCVA), intraocular pressure measurement, anterior segment evaluation by slit-lamp biomicroscopy, fundus examination by indirect ophthalmoscopy, OCTA scanning, flare-visional evoked potential (FVEP), and Orbital magnetic resonance image (MRI). All examinations were conducted by the same experienced clinician to ensure procedural consistency. Eyes were classified as affected (ON) or unaffected (NON) based on a documented history of ON, corroborated by clinical examination and ancillary tests.

### 2.2 Visual acuity assessment

Best-corrected visual acuity (BCVA) was assessed using a Snellen chart and then converted to the logarithm of the minimum angle of resolution (logMAR) scale for statistical analysis. Counting fingers was recorded as logMAR = 2.10, hand-motion perception as logMAR = 2.40, light perception as logMAR = 2.70, and no light perception as logMAR = 3.00 (13).

### 2.3 Spectral-domain OCT and OCTA scans

OCTA images were acquired by an experienced technician using swept-source optical coherence tomography (SS-OCT; VG 200D, SVision Imaging Ltd., Luoyang, China). All participants were scanned with the ganglion cell–inner plexiform layer (GCIPL) thickness protocol and the vascular -retina mode centered on the fovea (6 × 6 mm). Only scans with a quality index (QI) ≥ 7/10 were included in the final analysis. For patients with a history of MOG-ON/NMOSD, both eyes were included in the OCT and OCTA analyses.

Retinal microvascular and structural parameters were evaluated automatically using the built-in software(Vangogh software version 3.1.257). The primary outcomes included: microvascular density (MVD) of the superficial vascular complex (SVC) in the macula and the radial peripapillary capillary plexus (RPCP), vascular density (VD) of the SVC and deep vascular complex (DVC), and blood flow area (BFA) of the SVC, DVC, and choriocapillaris (CC). The SVC includes the RPCP and superficial vascular plexus (SVP), while the DVC comprises the intermediate capillary plexus (ICP) and deep capillary plexus (DCP). The boundary between the SVC and DVC was defined at the interface between the inner two-thirds and outer one-third of the GCIPL. VD was calculated as the percentage of area occupied by large vessels and microvessels, MVD as the percentage of area occupied by microvessels with diameters < 40 μm. And the BFA refers to the total area of pixels above the defined threshold in a binarized image. Image binarization is a process in which the grayscale values of all pixels are converted to either 0 or 255, resulting in an image composed solely of black and white regions.

### 2.4 Statistical analysis

Normally distributed continuous variables were expressed as mean ± SD and compared among groups using one-way analysis of variance (ANOVA). Non-normally distributed variables were expressed as median (IQR) and compared using the non-parametric Kruskal–Wallis H test. Categorical variables were expressed as percentages (%) and compared using the chi-square test; when expected cell counts were < 10, Fisher’s exact test was applied. Pairwise comparisons of OCT/OCTA parameters were adjusted using the Bonferroni correction. Correlations between OCT/OCTA metrics and BCVA, disease duration, and Expanded Disability Status Scale (EDSS) scores were analyzed using Spearman’s correlation. Data were processed with R (version 4.2.0; The R Foundation for Statistical Computing) and EmpowerStats (X & Y Solutions Inc, Boston, MA). A *P*-value < 0.05 was considered statistically significant.

## 3 Results

### 3.1 Demographic and clinical features of the subjects

Our study included 46 eyes from 23 individuals with MOGAD (mean age, 41.5 ± 15.5 years), 44 eyes from 22 individuals with NMOSD (mean age, 46.8 ± 14.1 years), and 40 eyes from 20 HCs(mean age, 45.4 ± 15.7 years). There were no significant differences in age or sex among the three subgroups (*P* > 0.05; **Table 1**). Compared with MOGAD, NMOSD showed poorer BCVA (*P* = 0.002) and higher EDSS scores (*P* < 0.001); however, no significant differences were found between the two groups in the number of ON episodes, antibody titer, or disease duration (*P* > 0.05; **Table 1**). Among included patients, fourteen NMOSD patients were bilateral affected and 12 MOGAD patients were bilateral affected. Therefore, 36 NMOSD-ON eyes, 8 NMOSD-NON eyes, 34 MOG-ON eyes and 12 MOG-NON eyes were conducted further OCT/OCTA analysis.

**Table 1:** Clinical characteristic of NMOSD, MOGAD and HCs.

Variables	NMOSD (n = 22)	MOGAD (n = 23)	HCs (n = 20)	*P*-value
age, Mean ± SD	46.8 ± 14.1	41.5 ± 15.5	45.4 ± 15.7	0.481
gender, n (%)				0.502
female	21 (95.5)	21 (91.3)	17 (85)
male	1 (4.5)	2 (8.7)	3 (15)
Affected side, n (%)				0.373
unilateral	8 (36.3)	12 (52.1)	-	
binocular	14 (63.6)	11 (47.8)	-	
EDSS, Median (IQR)	3 (2, 4)	1 (1, 2)	-	<0.001
BCVA logMAR, Median (IQR)	0.7 (0.1, 2.1)	0.2 (0.0, 0.3)	-	0.002
ON attack times, n (%)				0.551
1	8 (36.4)	9 (39.1)	-	
2	8 (36.4)	6 (26.1)	-	
3	4 (18.2)	2 (8.7)	-	
4	0 (0)	3 (13)	-	
5	1 (4.5)	1 (4.3)	-	
7	1 (4.5)	2 (8.7)	-	
Titer, n (%)				0.537
1:10	3 (15.79%)	4 (20.00%)	-
1:32	2 (10.53%)	5 (25.00%)	-
1:100	6 (31.58%)	6 (30.00%)	-
1:320	7 (36.84%)	5 (25.00%)	-
1:1000	1 (5.26%)	0 (0.00%)	-
Disease duration (months), Median (IQR)	33.3 (13.2, 66.5)	19.2 (7.1, 51.1)	-	0.586

Data are presented as mean ± SD, median (IQR), or n (%). NMOSD, neuromyelitis optica spectrum disorder; MOGAD, myelin oligodendrocyte glycoprotein antibody–associated disease; HCs, healthy controls; ON, optic neuritis; BCVA, best-corrected visual acuity; EDSS, Expanded Disability Status Scale.

### 3.2 Comparison of OCT/OCTA characteristics in the macular area among NMOSD-ON, MOG-ON, and HCs Eyes

**Figure 2:**
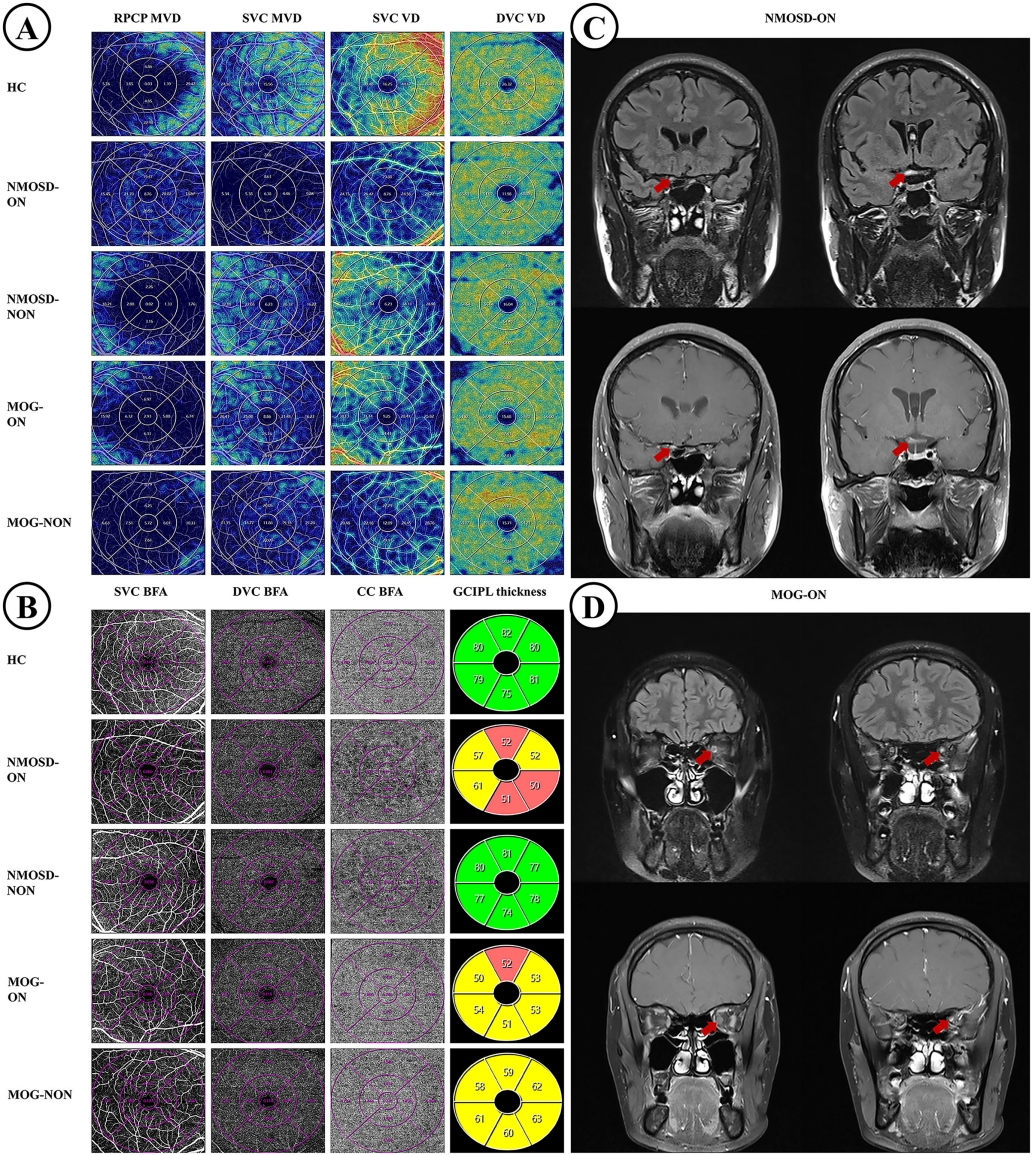
Comparison of OCTA microvascular characteristics and GCIPL thickness among HCs, NMOSD, and MOGAD patients. **(A, B)** show representative OCTA microvascular features and GCIPL thickness maps from the right eye of a 43-year-old female patient with NMOSD-ON and the left eye of an 18-year-old female patient with MOG-ON, respectively. **(C)** presents orbital magnetic resonance imaging (MRI) of the right eye in the NMOSD-ON patient, and **(D)** presents orbital MRI of the left eye in the MOG-ON patient. NMOSD, neuromyelitis optica spectrum disorder; MOG, myelin oligodendrocyte glycoprotein; HCs, healthy controls; ON, optic neuritis; NON, no optic neuritis; SVC, superficial vascular complex; RPCP, radial peripapillary capillary plexus; DVC, deep vascular complex; CC, choriocapillaris; GCIPL, ganglion cell–inner plexiform layer; MVD, microvascular density; VD, vascular density; BFA, blood flow area.

**Figure 3:**
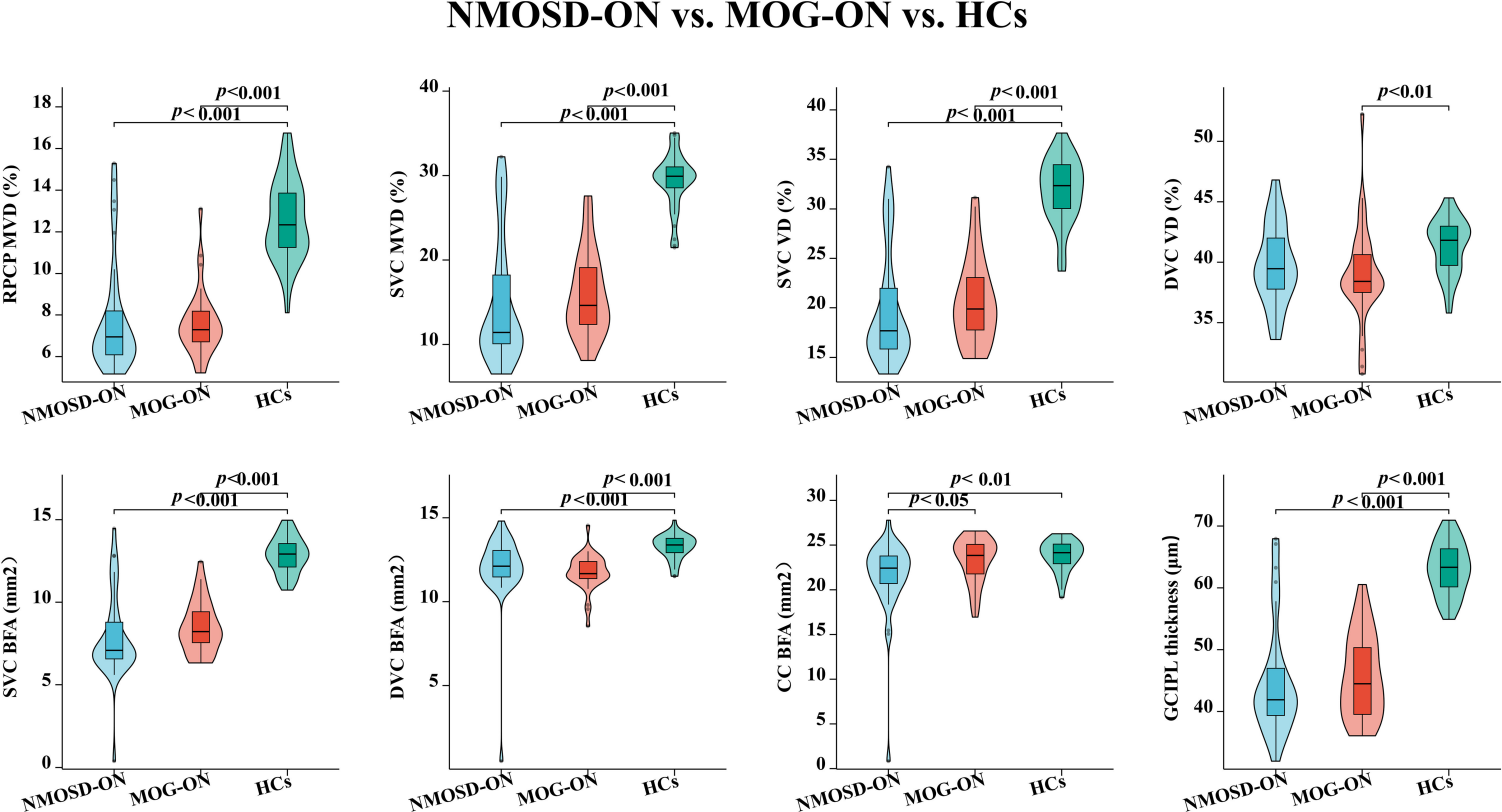
Comparison of OCTA microvascular characteristics and GCIPL thickness among NMOSD-ON, MOG-ON and HCs eyes. NMOSD, neuromyelitis optica spectrum disorder; MOG, myelin oligodendrocyte glycoprotein; HCs, healthy controls; ON, optic neuritis; SVC, superficial vascular complex; RPCP, radial peripapillary capillary plexus; DVC, deep vascular complex; CC, choriocapillaris; GCIPL, ganglion cell-inner plexiform layer; MVD, microvascular density; VD, vascular density; BFA, blood flow area.

Compared with HCs (29.41 ± 3.24%;12.58 ± 1.91%), both NMOSD-ON (15.00 ± 7.45%; 7.82 ± 2.68%) and MOG-ON (15.92 ± 4.96%; 7.59 ± 1.58%) eyes exhibited significantly reduced MVD in the SVC and RPCP (*P* < 0.001; **Table 2**; **Figures 2**, **3**). Similarly, the VD and BFA of the SVC were markedly lower in NMOSD-ON (19.83 ± 5.66%;7.89 ± 2.58 mm²) and MOG-ON (20.71 ± 4.27%; 8.50 ± 1.56 mm²) eyes than in HCs (31.82 ± 3.29%; 12.85 ± 1.09 mm²; *P* < 0.001; **Table 2**; **Figures 2**, **3**). Notably, both VD (38.99 ± 3.97%) and BFA (11.76 ± 1.07 mm²) in the DVC were significantly reduced in MOG-ON eyes compared with HCs (41.32 ± 2.27%; 13.27 ± 0.73 mm²; **Table 2**; **Figures 2**, **3**). In contrast, among NMOSD-ON eyes, only the BFA of the DVC (12.03 ± 2.23 mm²) showed a significant reduction relative to HCs (13.27 ± 0.73 mm²; *P* = 0.001; **Figure 3**).

**Table 2:** OCT/OCTA characteristics of macular area in patients with NMOSD-ON and MOG-ON compared with HCs eyes.

Variables	NMOSD-ON affected eye (n = 36)	MOG-ON affected eye (n = 34)	HCs (n = 40)	*P*-value	P1 (NMOSD-ON vs MOG-ON)	P2 (NMOSD-ON vs HCs)	P3 (MOG-ON vs HCs)
MVD (%)							
RPCP	7.82 ± 2.68	7.59 ± 1.58	12.58 ± 1.91	<0.001	0.892	<0.001	<0.001
SVC	15.00 ± 7.45	15.92 ± 4.96	29.41 ± 3.24	<0.001	0.760	<0.001	<0.001
VD (%)							
SVC	19.83 ± 5.66	20.71 ± 4.27	31.82 ± 3.29	<0.001	0.693	<0.001	<0.001
DVC	39.82 ± 3.29	38.99 ± 3.97	41.32 ± 2.27	0.008	0.528	0.106	0.006
BFA (mm^2^)							
SVC	7.89 ± 2.58	8.50 ± 1.56	12.85 ± 1.09	<0.001	0.341	<0.001	<0.001
DVC	12.03 ± 2.23	11.76 ± 1.07	13.27 ± 0.73	<0.001	0.717	<0.001	<0.001
CC	21.49 ± 4.43	23.31 ± 2.53	23.84 ± 1.70	0.004	0.040	0.003	0.738
Thickness (μm)							
GCIPL	44.86 ± 9.12	45.71 ± 6.73	63.10 ± 4.19	<0.001	0.862	<0.001	<0.001

NMOSD, neuromyelitis optica spectrum disorder; MOG, myelin oligodendrocyte glycoprotein; HCs, healthy controls; ON, optic neuritis; NON, no optic neuritis; SVC, superficial vascular complex; RPCP, radial peripapillary capillary plexus; DVC, deep vascular complex; CC, choriocapillaris; GCIPL, ganglion cell–inner plexiform layer; MD, microvascular density; VD, vascular density; BFA, blood flow area.

The BFA of the choriocapillaris (CC) in NMOSD-ON eyes (21.49 ± 4.43 mm²) was significantly lower than that in both MOG-ON (23.31 ± 2.53 mm²; *P* = 0.040) and HCs (23.84 ± 1.70 mm²; *P* = 0.003). No significant difference was observed between MOG-ON and HCs (*P* = 0.738; **Table 2**; **Figures 2**, **3**). All other OCTA parameters showed no statistically significant differences between the MOG-ON and NMOSD-ON groups (*P* > 0.05; **Table 2**; **Figures 2**, **3**).

Comparison of GCIPL thickness across the three groups revealed that both NMOSD-ON (44.86 ± 9.12μm) and MOG-ON (45.71 ± 6.73 μm) eyes demonstrated a pronounced reduction compared with HCs (63.10 ± 4.19 μm; *P* < 0.001; **Table 2**; **Figures 2**, **3**).

**Figure 2** Comparison of OCTA microvascular characteristics and GCIPL thickness among HCs, NMOSD, and MOGAD patients. Panels A and B show representative OCTA microvascular features and GCIPL thickness maps from the right eye of a 43-year-old female patient with NMOSD-ON and the left eye of an 18-year-old female patient with MOG-ON, respectively. Panel C presents orbital magnetic resonance imaging (MRI) of the right eye in the NMOSD-ON patient, and Panel D presents orbital MRI of the left eye in the MOG-ON patient.

NMOSD, neuromyelitis optica spectrum disorder; MOG, myelin oligodendrocyte glycoprotein; HCs, healthy controls; ON, optic neuritis; NON, no optic neuritis; SVC, superficial vascular complex; RPCP, radial peripapillary capillary plexus; DVC, deep vascular complex; CC, choriocapillaris; GCIPL, ganglion cell-inner plexiform layer; MVD, microvascular density; VD, vascular density; BFA, blood flow area.

**Figure 3** Comparison of OCTA microvascular characteristics and GCIPL thickness among NMOSD-ON, MOG-ON and HCs eyes. NMOSD, neuromyelitis optica spectrum disorder; MOG, myelin oligodendrocyte glycoprotein; HCs, healthy controls; ON, optic neuritis; SVC, superficial vascular complex; RPCP, radial peripapillary capillary plexus; DVC, deep vascular complex; CC, choriocapillaris; GCIPL, ganglion cell-inner plexiform layer; MVD, microvascular density; VD, vascular density; BFA, blood flow area.

### 3.3 Comparison of OCT/OCTA characteristics in the macular area among NMOSD-ON, NMOSD-NON, and HCs Eyes

**Figure 4:**
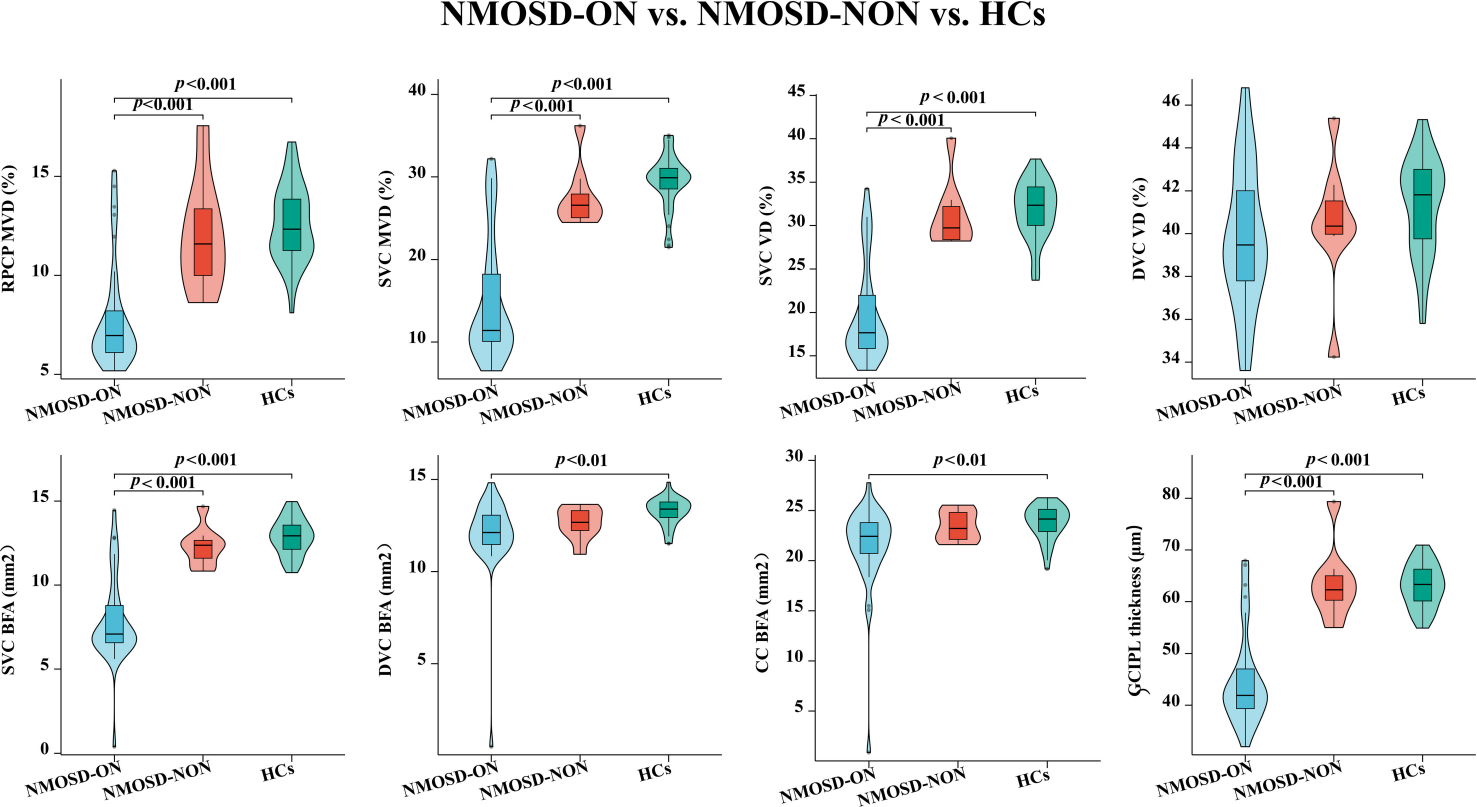
Comparison of OCTA microvascular characteristics and GCIPL thickness among NMOSD-ON, NMOSD-NON and HCs eyes. NMOSD, neuromyelitis optica spectrum disorder; HCs, healthy controls; ON, optic neuritis; NON, no optic neuritis; SVC, superficial vascular complex; RPCP, radial peripapillary capillary plexus; DVC, deep vascular complex; CC, choriocapillaris; GCIPL, ganglion cell-inner plexiform layer; MVD, microvascular density; VD, vascular density; BFA, blood flow area.

A total of 22 individuals with NMOSD (44 eyes) were included in the analysis. Based on the clinical history of optic neuritis, 36 eyes were classified as NMOSD-ON and 8 as NMOSD-NON (**Table 3**; **Figure 4**). In NMOSD-ON eyes, the MVD (15.00 ± 7.45%), VD (19.83 ± 5.66%), and BFA (7.89 ± 2.58 mm²) of the SVC; the MVD of the RPCP (7.82 ± 2.68%); and the GCIPL thickness (44.86 ± 9.12 μm) were all significantly reduced compared with both NMOSD-NON and HCs (**Table 3**; **Figure 4**). The BFA of the DVC (12.03 ± 2.23 mm²) and CC (21.49 ± 4.43 mm²) in NMOSD-ON eyes was significantly lower than that in HCs (13.27 ± 0.73 mm²; 23.84 ± 1.70 mm²) but showed no significant difference compared with NMOSD-NON (**Table 3**; **Figure 4**). No significant differences were observed in the VD of the DVC among the three groups (*P* = 0.073; **Table 3**; **Figure 4**). Furthermore, none of the OCT/OCTA parameters differed significantly between NMOSD-NON and HCs.

**Table 3:** OCT/OCTA characteristics of macular area in patients with NMOSD-ON, NMOSD-NON, and HCs eyes.

Variables	NMOSD-ON (n = 36)	NMOSD-NON (n = 8)	HCs (n = 40)	*P*-value	P1 (NMOSD-ON vs NMOSD-NON)	P2 (NMOSD-ON d vs HCs)	P3 (NMOSD-NON vs HCs)
MVD (%)							
RPCP	7.82 ± 2.68	12.04 ± 2.86	12.58 ± 1.91	<0.001	<0.001	<0.001	0.825
SVC	15.00 ± 7.45	27.63 ± 3.86	29.41 ± 3.24	<0.001	<0.001	<0.001	0.680
VD (%)							
SVC	19.83 ± 5.66	31.19 ± 3.99	31.82 ± 3.29	<0.001	<0.001	<0.001	0.930
DVC	39.82 ± 3.29	40.48 ± 3.10	41.32 ± 2.27	0.073	0.822	0.858	0.720
BFA (mm^2^)							
SVC	7.89 ± 2.58	12.33 ± 1.20	12.85 ± 1.09	<0.001	<0.001	<0.001	0.757
DVC	12.03 ± 2.23	12.64 ± 0.89	13.27 ± 0.73	0.004	0.588	0.002	0.551
CC	21.49 ± 4.43	23.42 ± 1.53	23.84 ± 1.70	0.007	0.270	0.005	0.939
Thickness (μm)							
GCIPL	44.86 ± 9.12	63.64 ± 7.30	63.10 ± 4.19	<0.001	<0.001	<0.001	0.978

NMOSD, neuromyelitis optica spectrum disorder; HCs, healthy controls; ON, optic neuritis; NON, no optic neuritis; SVC, superficial vascular complex; RPCP, radial peripapillary capillary plexus; DVC, deep vascular complex; CC, choriocapillaris; GCIPL, ganglion cell-inner plexiform layer; MVD, microvascular density; VD, vascular density; BFA, blood flow area.

**Figure 4** Comparison of OCTA microvascular characteristics and GCIPL thickness among NMOSD-ON, NMOSD-NON and HCs eyes. NMOSD, neuromyelitis optica spectrum disorder; HCs, healthy controls; ON, optic neuritis; NON, no optic neuritis; SVC, superficial vascular complex; RPCP, radial peripapillary capillary plexus; DVC, deep vascular complex; CC, choriocapillaris; GCIPL, ganglion cell-inner plexiform layer; MVD, microvascular density; VD, vascular density; BFA, blood flow area.

### 3.4 Comparison of OCT/OCTA characteristics in the macular area among MOG-ON, MOG-NON, and HCs Eyes

**Figure 5:**
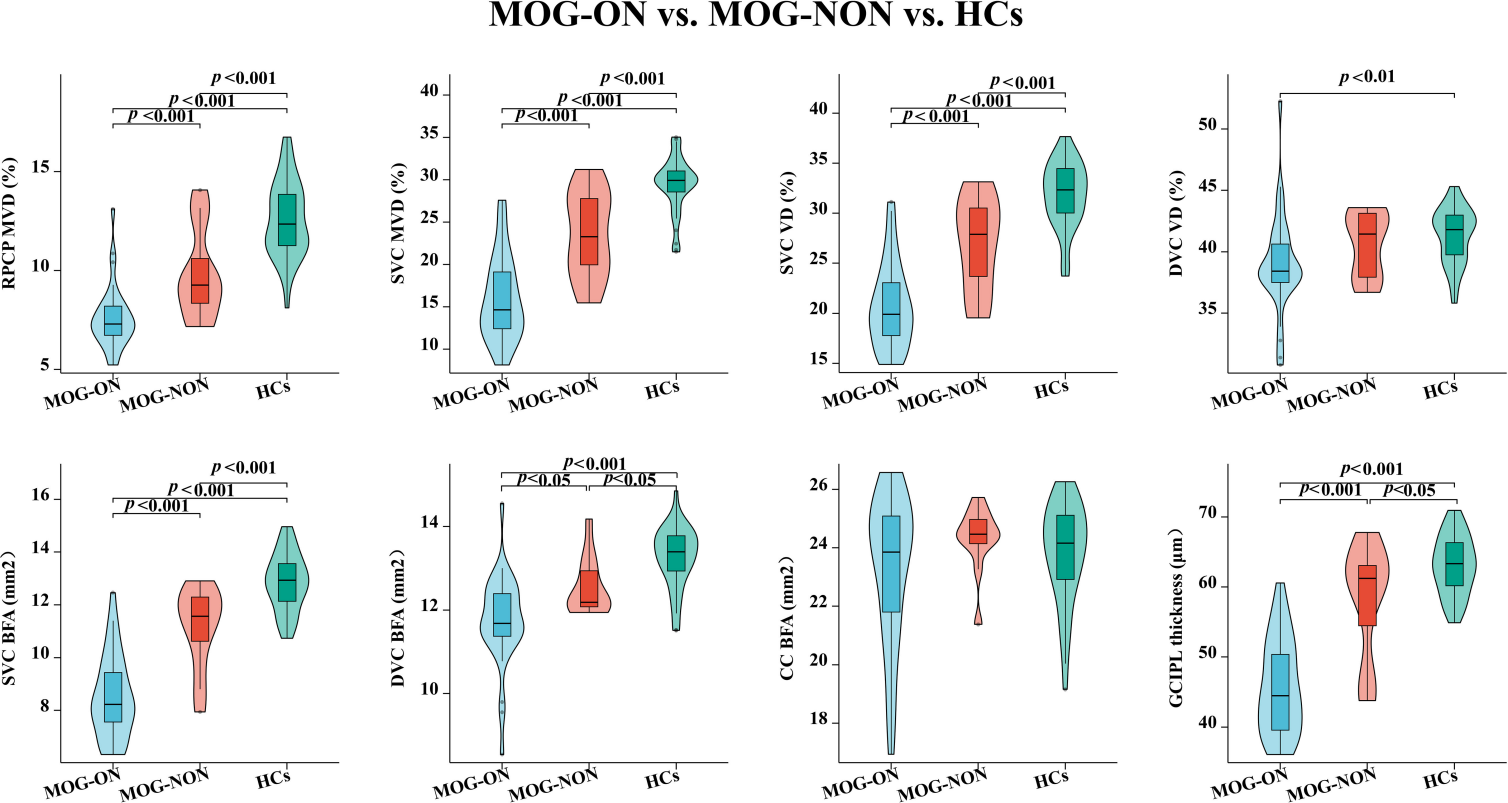
Comparison of OCTA microvascular characteristics and GCIPL thickness among MOG-ON, MOG-NON and HCs eyes. MOG, myelin oligodendrocyte glycoprotein; HCs, healthy controls; ON, optic neuritis; NON, no optic neuritis; SVC, superficial vascular complex; RPCP, radial peripapillary capillary plexus; DVC, deep vascular complex; CC, choriocapillaris; GCIPL, ganglion cell-inner plexiform layer; MVD, microvascular density; VD, vascular density; BFA, blood flow area.

A total of 23 individuals with MOGAD, comprising 34 MOG-ON eyes and 12 MOG-NON eyes, were included in the analysis (**Table 4**). Both MOG-ON and MOG-NON eyes showed significantly reduced MVD (15.92 ± 4.96%; 23.54 ± 5.15%), VD (20.71 ± 4.27%;26.91 ± 4.73%), and BFA (8.50 ± 1.56 mm²; 11.16 ± 1.55 mm²) of the SVC, as well as decreased MVD of the RPCP (7.59 ± 1.58%;9.79 ± 2.32%), compared with HCs (*P* < 0.001; **Table 4**; **Figure 5**). Further analysis revealed significantly greater reductions in these parameters in MOG-ON compared with MOG-NON eyes (*P* < 0.001; **Table 4**; **Figure 5**). Regarding the VD of the DVC, a significant reduction was observed exclusively in MOG-ON eyes (38.99 ± 3.97%) compared with HCs (41.32 ± 2.27%; *P* = 0.005; **Table 4**; **Figure 5**). For the BFA within the DVC, MOG-ON eyes exhibited the lowest values (11.76 ± 1.07 mm²), whereas HCs showed the highest (13.27 ± 0.73 mm²), with all pairwise comparisons revealing statistically significant differences. A similar trend was observed for GCIPL thickness across the three groups, whereas no significant differences were found in CC BFA among the groups (*P* > 0.05; **Table 4**; **Figure 5**).

**Table 4:** OCT/OCTA characteristics of macular area in patients with MOG-ON, MOG-NON, and HCs eyes.

Variables	MOG-ON (n = 34)	MOG-NON (n = 12)	HCs (n = 40)	*P*-value	P1 (MOG-ON vs MOG-NON)	P2 (MOG-ON vs HCs)	P3 (MOG-NON vs HCs)
MVD (%)							
RPCP	7.59 ± 1.58	9.79 ± 2.32	12.58 ± 1.91	<0.001	<0.001	<0.001	<0.001
SVC	15.92 ± 4.96	23.54 ± 5.15	29.41 ± 3.24	<0.001	<0.001	<0.001	<0.001
VD (%)							
SVC	20.71 ± 4.27	26.91 ± 4.73	31.82 ± 3.29	<0.001	<0.001	<0.001	<0.001
DVC	38.99 ± 3.97	40.63 ± 2.63	41.32 ± 2.27	0.007	0.262	0.005	0.774
BFA (mm^2^)							
SVC	8.50 ± 1.56	11.16 ± 1.55	12.85 ± 1.09	<0.001	<0.001	<0.001	<0.001
DVC	11.76 ± 1.07	12.55 ± 0.69	13.27 ± 0.73	<0.001	0.023	<0.001	0.037
CC	23.31 ± 2.53	24.31 ± 1.12	23.84 ± 1.70	0.279	0.302	0.497	0.756
Thickness (μm)							
GCIPL	45.71 ± 6.73	58.21 ± 8.29	63.10 ± 4.19	<0.001	<0.001	<0.001	0.037

NMOSD, neuromyelitis optica spectrum disorder; MOG, myelin oligodendrocyte glycoprotein; HCs, healthy controls; ON, optic neuritis; NON, no optic neuritis; SVC, superficial vascular complex; RPCP, radial peripapillary capillary plexus; DVC, deep vascular complex; CC, choriocapillaris; GCIPL, ganglion cell-inner plexiform layer; MVD, microvascular density; VD, vascular density; BFA, blood flow area.

**Figure 5** Comparison of OCTA microvascular characteristics and GCIPL thickness among MOG-ON, MOG-NON and HCs eyes. MOG, myelin oligodendrocyte glycoprotein; HCs, healthy controls; ON, optic neuritis; NON, no optic neuritis; SVC, superficial vascular complex; RPCP, radial peripapillary capillary plexus; DVC, deep vascular complex; CC, choriocapillaris; GCIPL, ganglion cell-inner plexiform layer; MVD, microvascular density; VD, vascular density; BFA, blood flow area.

### 3.5 Correlation between clinical features and OCT/OCTA characteristics in NMOSD-ON and MOG-ON groups

**Figure 6:**
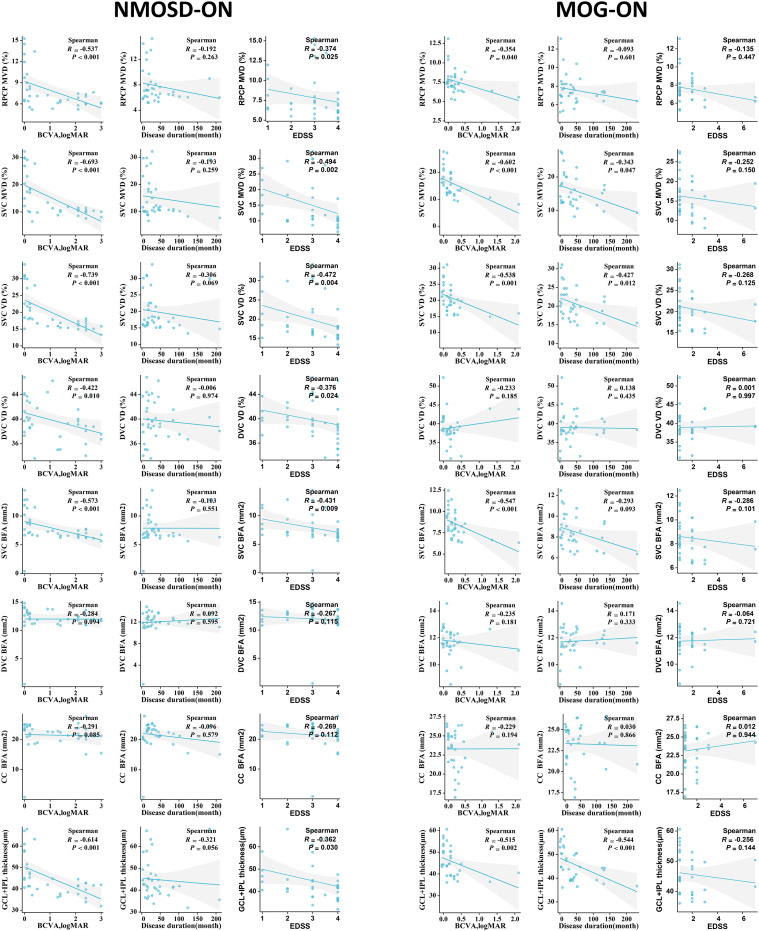
Correlation between clinical features and OCT/OCTA characteristics in NMOSD-ON and MOG-ON groups. NMOSD, neuromyelitis optica spectrum disorder; MOG, myelin oligodendrocyte glycoprotein; ON, optic neuritis; SVC, superficial vascular complex; RPCP, radial peripapillary capillary plexus; DVC, deep vascular complex; CC, choriocapillaris; GCIPL, ganglion cell–inner plexiform layer; VD, vascular density; BFA, blood flow area; BCVA, best-corrected visual acuity; EDSS, Expanded Disability Status Scale.

As shown in **Figure 6**, all MVD, VD, and GCIPL thickness values in NMOSD-ON eyes showed significant inverse correlations with both BCVA and EDSS (*P* < 0.05), whereas no significant associations were observed with disease duration (*P* > 0.05). The BFA of the SVC was also negatively correlated with BCVA (R = **–**0.573, *P* < 0.001) and EDSS (R = **–**0.431, *P* = 0.009). In contrast, the BFA**s** of the DVC and CC showed no significant correlations with BCVA, EDSS, or disease duration (*P* > 0.05).

Among MOG-ON eyes, MVD within the SVC was inversely correlated with both BCVA (R = **–**0.602, *P* < 0.001) and disease duration (R = **–**0.343, *P* = 0.047), while MVD of the RPCP was negatively correlated with BCVA alone (R = **–**0.343, *P* = 0.040**;** **Figure 6**). For VD, only the SVC demonstrated inverse correlations with BCVA (R = **–**0.538, *P* = 0.001) and disease duration (R = **–**0.427, *P* = 0.012**;** **Figure 6**). A similar pattern was observed for BFA, with the SVC showing a negative correlation with BCVA (R = **–**0.547, *P* < 0.001**;** **Figure 6**), whereas BFAs of the DVC and CC were not significantly associated with BCVA or disease duration (*P* > 0.05**;** **Figure 6**). GCIPL thickness was inversely correlated with both BCVA (R = **–**0.515, *P* = 0.002) and disease duration (R = **–**0.544, *P* < 0.001**;** **Figure 6**). All OCT/OCTA parameters in MOGAD showed no significant correlations with EDSS, ON attack times and antibody titers (**Figure 6**; **Supplementary Figures 1**, **2**).

**Figure 6** Correlation between clinical features and OCT/OCTA characteristics in NMOSD-ON and MOG-ON groups. NMOSD, neuromyelitis optica spectrum disorder; MOG, myelin oligodendrocyte glycoprotein; ON, optic neuritis; SVC, superficial vascular complex; RPCP, radial peripapillary capillary plexus; DVC, deep vascular complex; CC, choriocapillaris; GCIPL, ganglion cell-inner plexiform layer; VD, vascular density; BFA, blood flow area; BCVA, best-corrected visual acuity; EDSS, Expanded Disability Status Scale.

## 4 Discussion

In this study, both NMOSD-ON and MOG-ON eyes showed significantly reduced MVD, VD, and BFA in the SVC; reduced MVD in the RPCP; reduced BFA in the DVC; and decreased GCIPL thickness compared with HCs. Additionally, VD in the DVC in MOG-ON and BFA in the CC in NMOSD-ON were lower than those in HCs. We further observed that NMOSD-ON exhibited a significantly reduced CC BFA compared with MOG-ON, whereas no significant differences were identified in other parameters between the two groups. NMOSD-NON did not differ notably from HCs, whereas MOG-NON demonstrated structural and microvascular impairments in the macular region. Moreover, SVC metrics (MVD, VD, and BFA) and GCIPL thickness were negatively correlated with BCVA, disease duration, or EDSS.

We found that MVD, VD, and BFA in the SVC were significantly reduced in NMOSD-ON compared with both HCs and the NMOSD-NON group, whereas no significant differences were observed between NMOSD-NON and HCs. These findings for VD and BFA are consistent with previous studies (14–16). In contrast, among individuals with MOGAD, SVC MVD, VD, and BFA were diminished regardless of ON history, with even lower values observed in the ON group than in the NON group. Notably, similar reductions in SVC VD have also been reported in pediatric patients with MOGAD (17). The reduction in the SVC vascular network may be associated with neurovascular unit dysfunction and hypoperfusion secondary to inner -retinal structural atrophy (17–19). Notably, the SVP, representing the innermost layer of the neurovascular unit and closely associated with astrocytes, appears particularly vulnerable in ON, consistent with our observation of pronounced SVC impairment (20, 21).

The innermost layer of the choroid, located adjacent to Bruch’s membrane, consists of small diameter fenestrated capillaries collectively referred to as the CC. In our study, eyes with ON in NMOSD exhibited a significantly reduced BFA in the CC compared with both MOG-ON and HCs, whereas no significant difference was observed between MOG-ON and healthy eyes. Previous studies have reported that, in pediatric ON associated with MOG-IgG and NMOSD-IgG positivity, the VD of the CC did not differ significantly from that of HCs (17). However, changes in CC BFA in ON have not been reported in either adults or children. We hypothesize that the reduction in CC perfusion may be attributable to its distinct properties: the CC is the only vascular layer within the choroid that contains fenestrations permitting fluid exchange. Moreover, the anterior CC harbors mast cells and macrophages, creating a pro-inflammatory microenvironment that may predispose it to secondary immune-mediated inflammation following ON (22).

Except for VD in the DVC and BFA in the CC, all other MVD, VD, and BFA metrics, as well as GCIPL thickness, in MOG-NON eyes were lower than those in HCs, suggesting that even clinically unaffected eyes in individuals with MOGAD may exhibit structural and microvascular macular injury. In contrast, NMOSD-NON eyes showed no such impairment. A previous meta-analysis reported that GCIPL and INL thicknesses in NMOSD-NON eyes were reduced compared with HCs, with no significant difference between MOG-NON and NMOSD-NON eyes implying that GCIPL and INL thinning may also occur in MOG-NON eyes (23).Although these findings diverge slightly from our results, they consistently suggest that MOG-NON eyes undergo subtle macular structural and perfusion alterations. One plausible mechanism is that retinal ganglion cells, which are central nervous system neurons, have axons (optic nerves) myelinated by oligodendrocytes and lack regenerative capacity after injury (24).MOG is localized on the oligodendrocyte membrane, and MOG-IgG-mediated injury represents a principal pathogenic mechanism in MOGAD (25). This observation supports the hypothesis that the clinically unaffected eye in MOGAD may, in fact, be subclinically affected without overt manifestations (26, 27).

Analysis of VD and BFA in the DVC in individuals with NMOSD revealed that only eyes with a history of ON exhibited a significantly reduced BFA compared with HCs, consistent with previous reports (14–16).Among those with MOGAD, both ON and NON eyes showed lower BFA values than HCs; moreover, a significant reduction in VD was observed only in the MOG-ON group. These findings suggest that BFA may be more sensitive than VD in detecting DVC impairment and that MOGAD may cause more pronounced DVC damage than NMOSD.

Studies on retinal ischemic mechanisms have shown that the inner nuclear layer, which is primarily supplied by the DVC, is more vulnerable to mild retinal hypoperfusion and ischemia (28).Clinically, MOG-ON patients tend to experience more severe involvement and edema of the anterior optic nerve and its sheath, whereas NMOSD-ON predominantly affects the posterior optic nerve.^(6)^ We therefore hypothesize that the more pronounced VD reduction in the DVC of MOG-ON eyes may result from mechanical compression of the retinal vasculature due to anterior optic nerve or sheath edema, leading to compromised retinal arterial perfusion and secondary ischemia of the DVC (29).

In NMOSD-ON eyes, MVD, VD, and BFA of the SVC, as well as GCIPL thickness, exhibited significant negative correlations with both EDSS and BCVA. In contrast, among MOG-ON eyes, these parameters correlated negatively with BCVA and disease duration. Furthermore, the VD of the DVC in NMOSD-ON eyes was also inversely associated with BCVA and EDSS. These correlation patterns in NMOSD-ON are consistent with previous findings (16, 30).Yanlin et al. (31) demonstrated that VD in the SVP and DCP within the macular region of MOG-ON eyes was related to BCVA, EDSS, and disease duration; however, differences in macular segmentation methods between studies preclude direct comparison. The distinct correlation profiles observed between NMOSD-ON and MOG-ON likely reflect differences in their underlying pathophysiological mechanisms and patterns of structural and microvascular impairment within the macula. Specifically, in NMOSD-ON, macular alterations correlate strongly with EDSS, suggesting that early macular changes may serve as prognostic indicators of neurological disability. Conversely, in MOGAD, the stronger association with disease duration implies that therapeutic strategies should emphasize relapse prevention to mitigate cumulative microvascular and structural damage. Collectively, these findings underscore the potential of retinal imaging biomarkers as valuable clinical tools for delineating disease-specific features in NMOSD and MOGAD.

### 4.1 Limitations

This cross-sectional study focused exclusively on the structural and microvascular characteristics of the macula in individuals with NMOSD and MOGAD, and thus has some limitations. Because of its design, longitudinal changes in macular architecture and microvasculature over the course of disease progression could not be assessed. Moreover, only patients with a history of optic neuritis were included, precluding evaluation of how encephalitic or myelitic involvement might influence macular microvascular features. In addition, NMOSD is frequently associated with coexisting autoimmune diseases; however, the impact of autoimmune comorbidities was not specifically analyzed in the present study. Furthermore, the potential effects of different immunosuppressive treatment regimens on macular retinal structure and microvascular parameters were not evaluated in this study. The relatively small sample size and restricted demographic scope may also introduce selection bias, potentially masking subtle intergroup differences and attenuating the strength of observed correlations. Moreover, the predominance of female participants (95.5% in NMOSD; 91.3% in MOGAD) may reduce the generalizability of these findings to male patients because of potential sex bias. Our findings are preliminary and require validation in a multicenter cohort using the same OCTA device and computational software to ensure generalizability.

## 5 Conclusion

This study revealed that individuals with NMOSD exhibited higher EDSS scores and poorer BCVA than those with MOGAD. A more pronounced reduction in the BFA of the choriocapillaris (CC) within the macular region was observed in NMOSD-ON eyes compared with MOG-ON eyes, whereas other parameters MVD, VD, and GCIPL thickness showed no significant intergroup differences. Relative to HCs, MOG-NON eyes demonstrated reductions in MVD, VD, and BFA of the SVC, BFA of the DVC, and GCIPL thickness. In contrast, NMOSD-NON eyes exhibited no comparable abnormalities. In NMOSD-ON eyes, MVD, VD, and GCIPL thickness correlated negatively with both BCVA and EDSS. Among MOG-ON eyes, SVC MVD, VD, BFA, and GCIPL thickness were negatively associated with BCVA, with SVC MVD and VD also inversely correlated with disease duration.

## Data Availability

The original contributions presented in the study are included in the article/**Supplementary Material**. Further inquiries can be directed to the corresponding author/s.
