# Supplementary material for: Distinct macular structural and microvascular alterations differentiate neuromyelitis optica spectrum disorder from myelin oligodendrocyte glycoprotein antibody–associated disease in optic neuritis
Source: Front Immunol. 2026 Mar 10;17:1759144. doi: 10.3389/fimmu.2026.1759144 (PMC13010087; doi:10.3389/fimmu.2026.1759144)
Supplement: Supplementary file 1 [file DataSheet1.pdf]

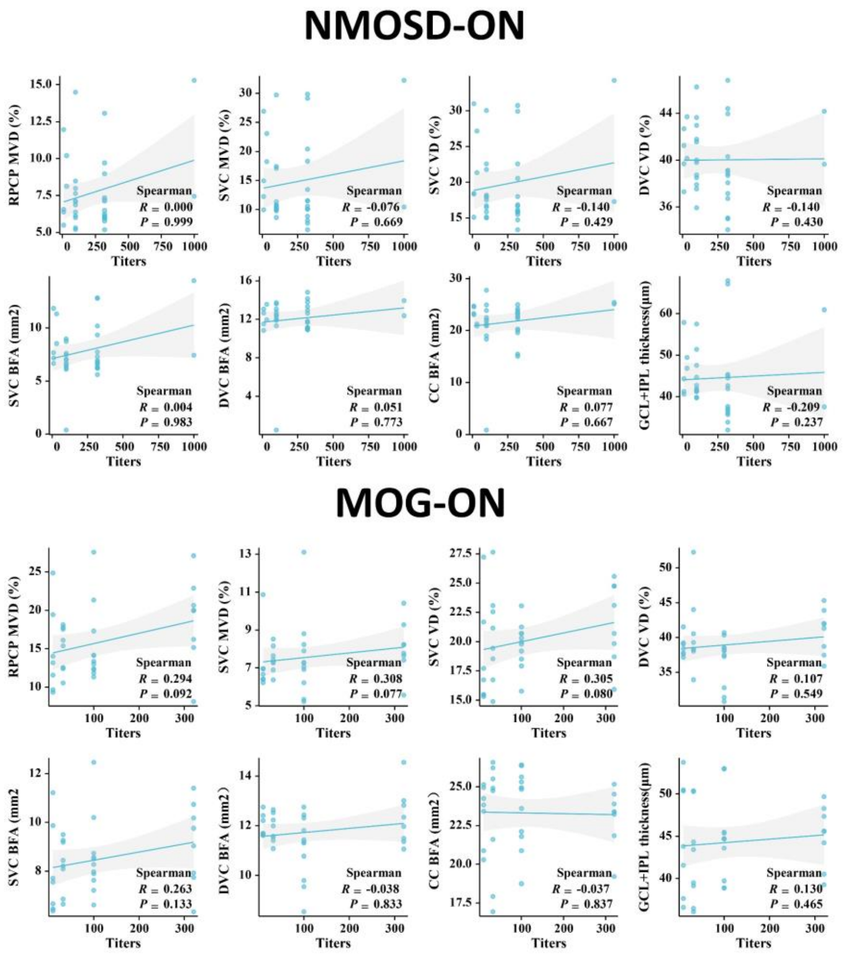

Supplementary Figure 1. Correlation between antibody titers and OCT/OCTA characteristics in NMOSD-ON and MOG-ON groups. NMOSD, neuromyelitis optica spectrum disorder; MOG, myelin oligodendrocyte glycoprotein; ON, optic neuritis; SVC, superficial vascular complex; RPCP, radial peripapillary capillary plexus; DVC, deep vascular complex; CC, choriocapillaris; GCIPL, ganglion cell–inner plexiform layer; VD, vascular density; BFA, blood flow area.
